# A Micro-CT Analysis of Initial and Long-Term Pores Volume and Porosity of Bioactive Endodontic Sealers

**DOI:** 10.3390/biomedicines10102403

**Published:** 2022-09-26

**Authors:** Mateusz Radwanski, Michal Leski, Adam K. Puszkarz, Jerzy Sokolowski, Louis Hardan, Rim Bourgi, Salvatore Sauro, Monika Lukomska-Szymanska

**Affiliations:** 1 Department of Endodontics, Medical University of Lodz, 251 Pomorska Str., 92-213 Lodz, Poland; 2 Institute of Material Science of Textiles and Polymer Composites, Faculty of Material Technologies and Textile Design, Lodz University of Technology, 116 Żeromskiego Street, 90-924 Lodz, Poland; 3 Department of General Dentistry, Medical University of Lodz, 251 Pomorska Str., 92-213 Lodz, Poland; 4 Department of Restorative Dentistry, School of Dentistry, Saint-Joseph University, Beirut 1107 2180, Lebanon; 5 Dental Biomaterials and Minimally Invasive Dentistry, Departamento de Odontología, Facultad de Ciencias de la Salud, Universidad CEU-Cardenal Herrera C/Del Pozo ss/n, Alfara del Patriarca, 46115 Valencia, Spain; 6 Department of Therapeutic Dentistry, I.M. Sechenov First Moscow State Medical University, 119146 Moscow, Russia

**Keywords:** bioactive sealers, initial porosity, long-term porosity, micro-computed tomography, pores volume

## Abstract

The evaluation of the porosities within the interface of root canals obturated with endodontics materials is extremely important for the long-term success of endodontic treatments. The aim of this study was to compare initial and long-term volume of pores (total, open, closed) and porosity (total, regional) of three bioactive endodontic sealers: GuttaFlow Bioseal, Total Fill BC Sealer, and BioRoot RCS. Root canals were obturated with three “bioactive” sealers using the single-cone technique. The volume of open and closed pores and porosity were calculated using a micro-computed tomography (MCT) method. The measurements were performed after 7 days (initial) and after 6 months (long-term) of incubation. Statistical significance was considered at *p* < 0.05. The total volume of pores remained unchanged after the 6-month storage. GuttaFlow Bioseal exhibited significantly higher long-term volume in open pores than Total Fill BC Sealer. The total porosity in all the tested sealers presented no statistically significant change after the 6-month storage, except for BioRoot RCS. The total porosity values of this latter material significantly increased after long-term incubation, especially in the apical region. In conclusion, the use of bioactive sealers with excessive tendency to create porosities both in shorth- and long-term periods of storage may compromise the long-term success of endodontic treatments.

## 1. Introduction

The root canal filling in endodontic treatments should provide a three-dimensional, fluid-tight seal of the prepared and disinfected space [1]. The most common material used for obturation is gutta-percha (GP), but due to the lack of adhesion to the root dentin, the application of a sealant is indispensable to achieve a suitable sealing at the interface [2]. Root canal sealers fill the space between the GP and the canal wall, flowing into lateral irregularities and accessory canals and spaces between GP points when used in a lateral compaction technique [3]. 

The first pre-blended and reusable calcium silicate sealant (CSBS), which was introduced in 2007 (iRoot SP, IBC, Burnaby, Canada), triggered significant development of these materials [4]. Today, CSBSs are available in a wide range of products varying in composition, properties, and consistency. CSBS requires water for the hydration reaction to ensure setting due to its hydraulic and hygroscopic properties [5]. The release of calcium and hydroxyl ions provides a high pH during setting and therefore a good anti-bacterial effect. In addition, the alkaline pH value is maintained for a long time, which favors the long-term elimination of bacteria [6,7]. Due to the chelation of calcium by ethylenediaminetetraacetic acid (EDTA), this solution is not recommended as a final rinse solution before obturation; it interferes with the hydration of the calcium silicate [1,8]. On the other hand, sodium hypochlorite (NaOCl) provides an alkaline pH, which may support the hydration and sealing ability [1,8]. Moreover, these materials can chemically bond to root canal dentin [5,6,9,10]. The exact mechanism is unknown; however, some concepts have been provided in the literature [11,12,13,14,15]. For instance, the formation of mechanical interlocking due to the diffusion of sealant particles into dentinal tubules was hypothesized [11]. A further theory assumed the formation of a mineral infiltration zone, which is associated with infiltration of the sealant mineral constituent into the inter-tubular dentin, as a consequence of the collagen fibers denaturation caused by a strongly alkaline sealant [12,13]. Bonding to the dentin can result from the formation of hydroxyapatite along the mineral infiltration zone due to the partial reaction of phosphate with calcium silicate hydrogel and calcium hydroxide [14,15]. It was reported that CSBSs are more biocompatible, showing less toxicity, than traditional sealants (e.g., AH Plus; Dentsply Sirona Endodontics, Ballaigues, Switzerland) [16,17,18]. They are also bioactive, positively influencing cellular interactions [19] and promoting osteoblast differentiation and deposition of cells necessary for wound healing [18]. These sealers can be used in both cold and warm root canal filling methods. However, the use of CSBS along with thermal techniques is still under dispute [10,11]. The application of heat extends the setting time of single-syringe materials and shortens the setting time of manually mixed ones [10,11]. In addition, the increase in temperature may cause the evaporation of water necessary to initiate the sealant setting process [10]. Additionally, these materials can be difficult to remove in case of re-treatment due to the hardness after setting. 

Silicone-based sealers, introduced to endodontics in 1972, are available in various forms, e.g., capsules or self-mixing syringes [20]. They set by addition reaction-forming polymer [20,21]. These materials are characterized by a good adaptation and homogeneity, and due to their viscosity and elasticity, they may absorb the stresses generated by mastication [22]. Some of these materials may contain bioactive glasses that induce precipitation of hydroxyapatite and/or calcium silicate, which, in turn, are responsible for the alkaline pH and the release of calcium ions, thus contributing to the regeneration and healing of bone and periapical tissues [23]. These latter sealants are designed only for the cold-filling method.

It is extremely important to eliminate the bacterial flora from the canal during endodontic treatment [24]. However, some areas of the canal space often remain untouched during chemo-mechanical preparation, regardless of the technique and files used [25]. Bacteria located in areas such as isthmuses, ramifications, deltas, and dentinal tubules may sometimes not be disinfected properly. Another important issue is dentin–sealer adaptation, known as a key factor yielding micro-leakage and root canal re-infection [26]. The re-colonization can occur with bacterial survival (biofilm) originating from coronal regions, inside the canal space, or outside the canal or infected soft tissue [27]. The single-cone technique is the recommended method of canal filling using both types of sealers [21,23,28,29]. It requires a GP cone of a size and taper matching the final instruments used for the canal preparation. Consequently, greater volume of the sealant is needed, which can result in the formation of air bubbles and pores [30,31]. The formation of pores contributes to the persistence of microbial infection in the root canal system and therefore is reported as a major factor associated with endodontic failure [32]. If the root canal filling does not provide a complete seal, the seepage of tissue fluids may become a substrate for bacterial growth [26,32].

The increasing number of pores over time can be associated with dissolution of the sealant [27]. Therefore, it is important to assess long-term changes in the porosity of sealants. Thus, the aim of this study was to compare initial and long-term volume of pores (total, open, closed) and porosity (total, regional) of three endodontic sealers: two bio-ceramics, Total Fill BC Sealer (FKG Dentaire, La-Chaux-de-Fonds, Switzerland) and BioRoot RCS (Septodont, Saint Maur Des Fosses, France), and one silicone-based sealer, GuttaFlow Bioseal (Coltène/Whaledent AG, Altstätten, Switzerland). The null hypothesis was that there would be no differences between the three tested sealers in terms of the evaluated parameters and no change in both parameters evaluated over time.

## 2. Materials and Methods

### 2.1. Sample Preparation 

After research ethics committee approval (RNN/36/20/KE; Lodz, Poland; 11/02/2020), 30 roots of freshly extracted first and second mandibular molars were included into the study. After manual debridement of the root surface with a curette, samples were disinfected in 5.25% NaOCl for 2 h and then stored in 0.5% thymol until experimentation. The sample size was calculated with the following parameters: effect size of 10%, standard deviation of 7%, significance level of 0.05, and power of 80%. The minimum sample size of 9 was determined.

The selection of the canals to be included in the study was performed based on a preliminary micro-computed tomography (MCT) analysis to provide homogeneity of evaluated roots in terms of anatomy. The following morphological parameters were considered: single, oval, canal in fully formed root, and curvatures between 10° and 20° calculated by Schneider’s method [33]. Additionally, roots with calcifications and internal or external resorption were excluded from the study. Moreover, the clinical inclusion criteria considered lack of root caries and an initial foramen diameter of canal equivalent to a size of 15 K-file (Dentsply Sirona Endodontics, Ballaigues, Switzerland).

The crowns were removed using a diamond bur (4ZR.FG.012; Komet Dental Gebr. Brasseler GmbH & Co., Lemgo, Germany) under continuous water-cooling, leaving 13 ± 1 mm long roots. A size 10 K-file (Dentsply Sirona Endodontics, Ballaigues, Switzerland) was inserted into the canals until the tip was visible at the apical foramen under the microscope (8X, OPMI Pico; Carl Zeiss, Oberkochen, Germany) and measured in mm after removal. The working length (WL) amounted to the above-mentioned value reduced by 1 mm.

The canals were instrumented by one operator using the X-smart Endodontic Motor (Dentsply Sirona Endodontics, Ballaigues, Switzerland) according to the manufacturer’s instructions. A glide path was created using several PathFiles (Dentsply Sirona Endodontics, Ballaigues, Switzerland) up to size 19, 0.02 taper. Apical patency was controlled with 10 K-file. The ProTaper Next (Dentsply Sirona Endodontics, Ballaigues, Switzerland) files were used with a constant speed of 300 revolutions per minute (rpm) and torque of 2 Ncm. The sequence was as follows: X1 (size 17, 0.04 taper), X2 (size 25, 0.06 taper), X3 (size 30, 0.07 taper), and X4 (size 40, 0.06 taper) up to the established WL. Each set (X1–X4) of rotary instruments was used for shaping one canal and then discarded. After using each file, copious irrigation with 5 mL of 5.25% NaOCl (CHLORAXiD, Cerkamed, Stalowa Wola, Poland) was performed for 120 ± 10 s. As a final rinse, canals were irrigated with 2.5 mL physiological saline and then with 5 mL 17% EDTA (Cerkamed, Stalowa Wola, Poland) for 1 min., 2.5 mL physiological saline, and 5 mL 5.25% NaOCl, followed by 2.5 mL of physiological saline. For irrigation, a 5 mL disposable plastic syringe with a 30-gauge Endo-Eze Tip (Ultradent, South Jordan, UT, USA) inserted without binding 2 mm shorter than WL was used. The solutions were manually activated with ProTaper Next X4 GP cone (Dentsply Sirona Endodontics, Ballaigues, Switzerland) using gentle strokes at a 100 times/minute cycle for 2 min. Finally, the canals were dried with paper absorbent points X4 (Dentsply Sirona Endodontics, Ballaigues, Switzerland).

In all study groups, canals were obturated using the single-cone technique with matched GP cones (40/0.06; Dentsply Sirona Endodontics, Ballaigues, Switzerland) and standardized amount of sealer (0.30 ± 0.010 g). The teeth were coded and randomly distributed into three groups (*n* = 10) based on the root canal sealers used: GuttaFlow Bioseal (Coltène/Whaledent AG, Altstätten, Switzerland), Total Fill BC Sealer (FKG Dentaire, La-Chaux-de-Fonds, Switzerland), and BioRoot RCS (Septodont, Saint Maur Des Fosses, France) (Table 1). The sealants were applied on the selected GP cone. The GP cone was covered with a standardized amount of sealer and slowly introduced into canal. Then, it was slowly and gently rotated (twice) to spread the sealer on the canal walls. Next, the GP cone was delicately pushed toward the apex to achieve the tag back and the estimated WL. The excess of material was removed using a hot plugger (Fast Pack Plugger Tips, size Fine Medium (Yellow) 50/0.05 taper, E-Connect Eighteenth, China), and the teeth were cleaned with isopropyl alcohol and temporarily filled with glass ionomer Fuji IX (GC Europe, Leuven, Belgium) to seal the canal. All specimens were stored in a Hank’s balanced salt solution (HBSS) at 37 °C for up to 6 months. The storage medium was renewed every 7 days.

### 2.2. Micro-CT Imaging

High-resolution MCT (SkyScan 1272; Bruker, Kontich, Belgium) scans were carried out under the following scanning conditions: X-ray source voltage, 90 kV; X-ray source current, 111 µA; and pixel size, 21 µm. Rotation of 180° was performed with a rotation step of 0.5°, and a copper + aluminum filter was selected. All specimens were scanned at four point times. Scan 1 was performed on the intact root canal for statistical analysis of volume variations between evaluated canals, while scan 2 occurred after root canal preparation with rotary files to measure the pre-obturation root canal volume. Initial pore volume and porosity after 7 days were evaluated on scan 3, whereas long-term pore volume and porosity after 6 months were evaluated on scan 4. 

**Figure 1:**
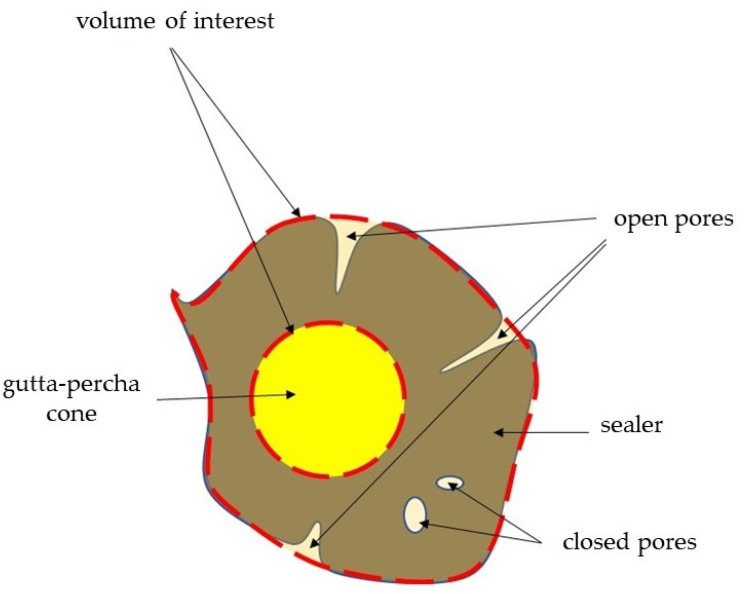
Scheme of volume of interest (VOI) cross-section.

**Figure 2:**
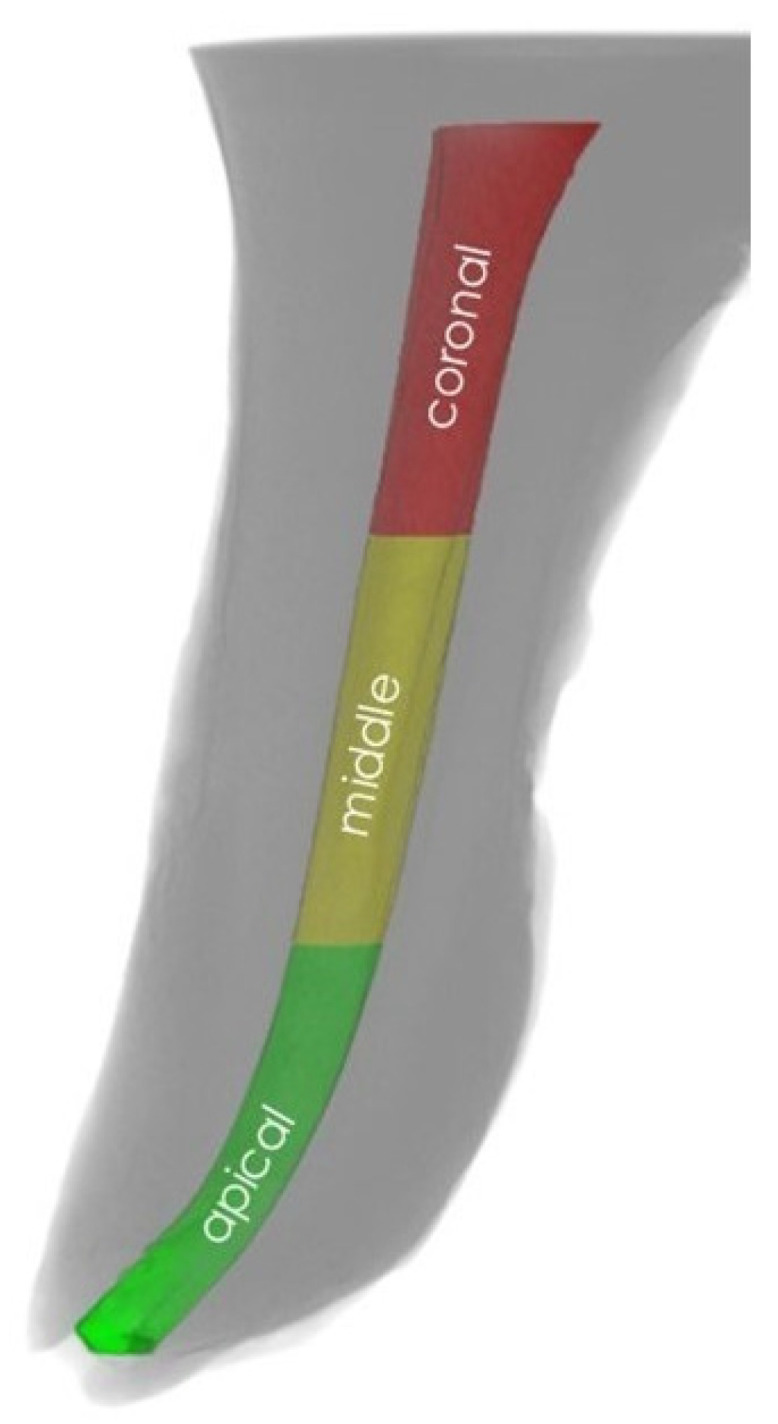
Volume of interest divided into three regions.

The images were re-constructed using NRecon v.1.6.0 software (Bruker, Kontich, Belgium), and the parameters were calculated using CTAn v1.14.4 software (Bruker, Kontich, Belgium). Three-dimensional (3D) visualization was achieved using CTvol v2.3.2.0 software (Bruker, Kontich, Belgium). The CTAn software enables the selection of the region of interest (ROI) based on the contrast of various image areas resulting from the un-like value of X-ray absorption by elements of scanned object due to difference in density. Namely, the X-ray absorption of the tooth differs from the X-ray absorption of the filling material, facilitating the selection of evaluated elements of scanned object: the cone absorbed X-rays more than the sealant, while the surrounding air absorbed X-rays less than the sealant. However, to ensure the measurement of all the open pores in the sealant, the ROI ran tangent to the sealant boundary (Figure 1). A volume of interest (VOI; mm^3^) was established from the furcation level to the apex of the root, and canals were divided into three equal regions: coronal, middle, and apical (Figure 2). The evaluation of MCT images was performed by a blinded researcher.

#### 2.2.1. Initial and Long-Term Volume of Pores 

**Figure 3:**
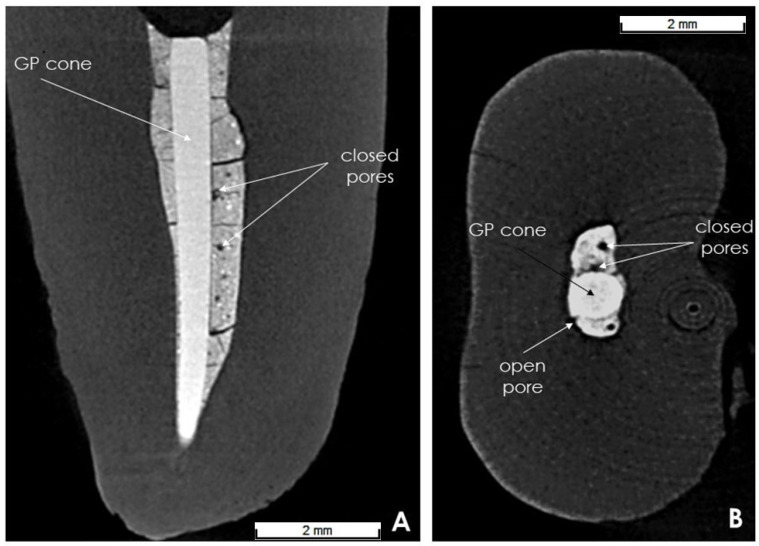
Representative transverse (**A**) and sagittal (**B**) cross sections illustrating the closed and open pores.

The initial total volume of pores was determined by subtracting the filling material volume after 7 days (scan 3) from the pre-obturation root canal volume (scan 2). Accordingly, long-term total volume of pores was calculated subtracting the filling material volume after 6 months (scan 4) from intact root canal volume (scan 2). On the one hand, closed pores were defined as not communicating with the root dentin and completely closed in the material (in the sealer or between the gutta-percha cone and the sealer) (Figure 3A,B). Conversely, open pores were defined as communicating with the dentin surface (located between the sealer and dentin wall) (Figure 3B). The volume of open and closed pores was calculated analogously for total pores volume. The change in pore volume (total, open, closed) over time was calculated by subtracting the initial volume (7 days) from the long-term volume (6 months). 

#### 2.2.2. Initial and Long-Term Porosity

Porosity refers to the percentage (%) of pore volume in relation to total sealer volume and was calculated for two time periods: initial (after 7 days, scan 3) and long-term (after 6 months, scan 4). The total porosity refers to the entire canal, while the regional porosity refers to root regions of equal length: coronal, middle, and apical. The change in porosity over time was calculated by subtracting the initial porosity (7 days) from the long-term porosity (6 months). 

### 2.3. Statistical Analysis 

The Shapiro–Wilk test was used to confirm the normality of the data. The analysis of variance was conducted with Levene’s test. The Student’s *t*-test and Friedman test were used for paired samples comparison. Moreover, Kruskal–Wallis’s test was performed for independent samples. All statistical analyses were evaluated with the statistical software package Statistica v. 13.1 (StatSoft, Inc., OK, USA), and statistical significance was considered at *p* < 0.05.

## 3. Results

### 3.1. Initial and Long-Term Volume of Pores

There was no significant difference in volumetric variance between prepared canals in the three study groups. 

Moreover, no statistically significant difference was found between the experimental groups regarding initial volumes of pores (open, closed, and total; *p* > 0.05) (Table 2). Nevertheless, after 6 months (long-term), the volume of open pores was significantly higher for GuttaFlow Bioseal compared with Total Fill BC Sealer (*p* < 0.05) (Table 3). In all groups, the mean percentage of open pores increased (after 6 months), in particular for GuttaFlow Bioseal; however, the difference was not statistically significant (*p* > 0.05). Moreover, there were no statistically significant differences in terms of initial and long-term total pore volumes between study groups (*p* > 0.05). The long-term total volume of pores decreased for GuttaFlow Bioseal and Total Fill BC Sealer, while it increased for BioRoot RCS, and such differences were statistically insignificant (*p* > 0.05).

**Figure 4:**
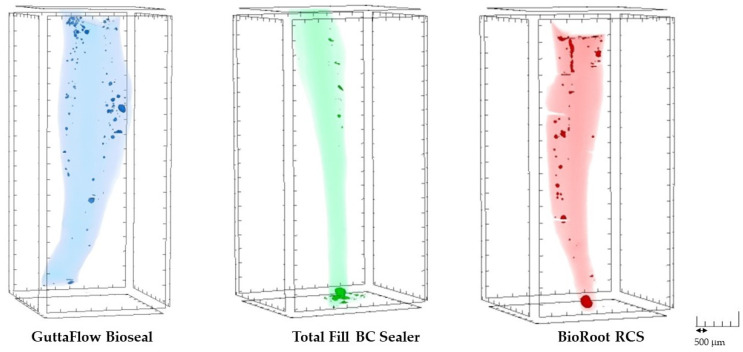
The three-dimensional (3D) reconstructions of the fillings of the random samples demonstrating different size and distribution of the pores through the filling materials.

The representative 3D images of pores distribution after 7-day incubation are presented in Figure 4.

### 3.2. Initial and Long-Term Porosity

**Figure 5:**
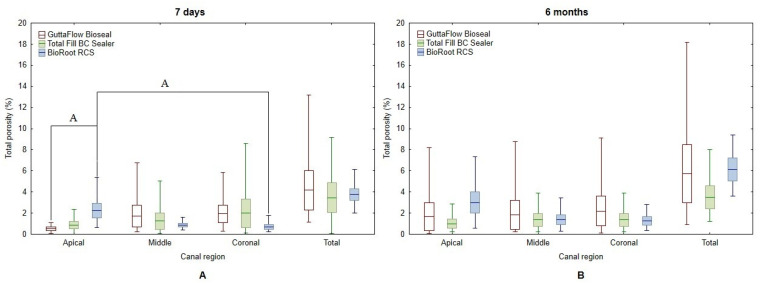
Regional and total porosity (%) of sealers in the root canal after 7 days (**A**) and 6 months (**B**). A—same superscript letters indicate statistically significant differences (*p* < 0.05).

For BioRoot RCS, the long-term total porosity was significantly higher than the initial one (*p* < 0.05). The highest change in total porosity (long-term vs. initial) was observed for BioRoot RCS (2.35% ± 2.20%), followed by GuttaFlow Bioseal (1.54% ± 3.91%), and the least was for Total Fill BC Sealer (0.02% ± 4.80%) (Figure 5); however, such differences were not statistically significant (*p* > 0.05).

After a 7-day incubation, significantly higher regional porosity was found in the apical part compared to the coronal area for BioRoot RCS (*p* < 0.05). In the apical region, a significantly higher porosity was found for BioRoot RCS compared to GuttaFlow Bioseal (*p* < 0.05). After 6 months, no statistically significant differences in regional porosity between the root regions were detected (Figure 5). 

The highest (both initial and long-term) apical porosity was observed for BioRoot RCS (2.24% ± 1.67% and 2.99% ± 2.46%, respectively). The smallest apical porosity after 7 days was detected in the specimens treated using GuttaFlow Bioseal (0.5% ± 0.4%), and after 6 months, in the specimens treated using Total Fill BC Sealer (1.00% ± 1.03%). The highest change in apical porosity was observed for GuttaFlow Bioseal (1.15% ± 3.23%), followed by BioRoot RCS (0.91% ± 1.11%) and by Total Fill BC Sealer (0.15% ± 0.47%), but these differences were not statistically significant (*p* > 0.05).

## 4. Discussion

The null hypothesis was rejected because significant differences were found between tested sealers in terms of the evaluated parameters initially and over time.

The main purpose of root canal filling is to prevent micro-leakage and root canal re-infection. Root canal treatment aims to prevent the formation of periapical lesions or, if they exist, facilitate their proper healing. Interestingly, the obturation technique and materials employed in the treatment were found to exert no influence on the prognosis of apical periodontitis [34]. However, this statement should be considered with caution due to the low number of included studies (10) into the meta-analysis and a high risk of bias. Therefore, further research is needed to investigate safety and success rate of obturation materials and techniques.

In the present study, three root canal sealants were selected for the analysis: two bio-ceramics (BioRoot RCS, manually mixed powder with liquid, and Total Fill BC Sealer, pre-mixed sealer), and a silicone-based, ready-to-use sealant (GuttaFlow Bioseal). These products were chosen for the study because they are modern and popular materials differing in composition and form of preparation. Contradictory classifications of GuttaFlow Bioseal can be found in the literature: due to the addition of calcium silicate, it is sometimes referred to as bio-ceramic sealer [35,36,37]. However, sealants should be classified according to the main components within their compositions, which in this case is a silicone; therefore, GuttaFlow Bioseal should be classified as silicone-based material [7,23,38]. The addition of substances, i.e., glass ceramic and/or calcium silicate, provides bio-active properties; thus, these sealers are considered bio-active [39]. Moreover, pre-mixed and syringe-mixed, with the ratio of ingredients controlled by the manufacturer, and manually mixed materials can be distinguished. The latter form do not provide a reproducible, faster, and cleaner mixing of the material [40].

The quality of a root canal filling in in vitro studies can be assessed with dye penetration, dye extraction (dissolution method) or fluid filtration (transportation method), glucose penetration model using fluid filtration, protein micro-leakage test, bacteria and toxin infiltration method, electrochemical micro-leakage test, radioisotope penetration method, microscopy, or 3D imaging (Cone-Beam Computed Tomography, MCT) [41,42]. Most of above-mentioned techniques lack quantitative measurements and may lead to sample destruction. Conversely, MCT is currently considered the most reliable non-destructive method for assessing the quality of filling over time. It allows for distinguishing between gutta-percha, sealant, and voids [9,43]. Moreover, the evaluation of voids/porosity in different parts of the root (apical, middle, coronal) and differentiation in the type of pores (open, closed) are feasible [5,9,43]. However, disadvantages include the high cost of this investigation [43]. 

The presence of pores was most frequently analyzed due to the space in which bacteria can re-grow and proliferate, causing long-term treatment failures [9,26,28,31,44,45]. Unfortunately, none of the filling materials or application methods can provide a pore-free root canal filling [9,45,46]. It is important to know whether pores are open or closed in the material, because it can determine the clinical success of the treatment. Indeed, open pores can cause micro-leakage, while closed ones seem to exert no clinical impact [46,47,48]. However, the presence of closed pores as well as their percentage was considered in the present study because they may turn into open ones with time, due to the sealant dissolution, causing a micro-leakage of clinical significance. Many different factors may influence the number of pores and porosity, including the filling technique, such as the wettability and flowability of the sealant; the form of the sealer (pre-mixed or manually mixed); irrigation protocol; and the anatomy of root canal [5]. 

In all studied groups (both initial and long-term), the mean volume and percentage of closed pores were greater than those of open ones. This finding is not supported in any other studies [9,31,49]. These differences may be explained by different anatomy of the examined roots in this study (distal roots of mandibular first molars), while others evaluated maxillary [9] or mandibular central incisors [49] or single-rooted pre-molars [31]. Additionally, the selection of shaping system: rotary or reciprocating and final instrument tip size and taper may exert an impact on the results. In the present study, ProTaper Next in a single length technique up to X4 (size 40, 0.06 taper) was applied. However, other studies used the crown-down technique along with rotary instruments: Reciproc R40 (size 40, 0.06 taper) (VDW, Munich, Germany) [31], Twisted Files Adaptive ML3 (size 50, 0.04 taper) (SybronEndo, Glendora, CA) [9], and EndoSequence (Brasseler, Savannah, GA) size 40 and 0.06 taper as a finishing files [49]. Moreover, the penetration of the sealant into the canal dentinal tubules and walls, and thus the presence of open pores, can be influenced by the irrigation protocol [50,51]. The application of chelating compounds immediately after NaOCl may contribute to dentin erosion, thereby reducing the adhesion surface of sealant [50,51]. However, the use of intermediate pH-neutral rinsing solution prevents the chelator inactivation caused by the oxidizing potential of NaOCl. As a result, the smear layer is being removed and the risk of micro-leakage minimized [31]. In the present study, physiological saline was applied as intermediate rinsing solution, while other studies used 17% EDTA (chelator) directly after 1% [9] or 5.25% [49] NaOCl [9,49]. The latter procedure contributed to dentinal erosion and insufficient removal of the smear layer and therefore insufficient sealer penetration into the canal walls and/or micro-leakage, increasing the open pores formation. In contrast, Angerame et al. [31] used distilled water as the intermediate rinsing solution. The application of EDTA (chelator) as the last rinsing solution before obturation can impar the hydration of the calcium silicate due to calcium chelation; this may have interfered with the setting process of bio-ceramic sealers [1,8].

Moreover, the mean long-term percentage of open pores in all tested groups increased when compared to initial one, and it was statistically higher for GuttaFlow Bioseal than for Total Fill BC Sealer (*p* < 0.05). However, the pore volume for GuttaFlow Bioseal was not previously evaluated; hence, it was not possible to compare the present findings with the results of previous studies. It can be hypothesized that higher detection of open pores is related to higher solubility of this material and/or weaker bonding to the dentin walls when compared to Total Fill BC Sealer. The greater solubility of GuttaFlow Bioseal in comparison to other sealants was observed in the study conducted by de Camargo et al. [52]. 

The release of calcium ions over time can contribute to the formation of precipitates that can accumulate in pores, thereby reducing their number over time [53]. Such a decrease in the number of pores may be associated with improved sealing ability, thus reducing the risk of micro-leakage and its clinical implications, i.e., development or persistence of periapical inflammation leading to treatment failure [28]. In the present study, after 6 months, the total volume of pores decreased for GuttaFlow Bioseal and Total-Fill BC Sealer, while they increased for BioRoot RCS. However, both differences (long-term vs. initial) were not statistically significant (*p* > 0.05). These results for Total Fill BC Sealer and BioRoot RCS are in agreement with previous studies [9]. However, a statistically significant increase in total volume of pores after 8 weeks of storage in a phosphate-rich medium for BioRoot RCS was observed by Atmeh et al. [28].

Porosity and other defects in the micro-structure of the endodontic sealant can cause foci of structural weakness and the tensile strength of the material, creating micro-cracks, which, in turn, can cause leakage within the endodontic cement in the root canal [54]. The total porosity changes for GuttaFlow Bioseal and Total Fill BC Sealer increased insignificantly over time (*p* > 0.05). However, the long-term total porosity of BioRoot RCS was significantly higher than initial one (*p* < 0.05). Similar results for Total Fill BC Sealer and BioRoot RCS were observed by Milanovic et al. [9], but the difference was statistically insignificant. The increase in porosity may be attributed to the enhanced solubility of bioactive sealers due to ion release [55,56]. In addition, the form and method of mixing sealers can affect the porosity and the presence of pores. Manually mixed materials are more prone to subjective factors induced by the operator, thus producing more structural defects [54,57,58]. It can be the reason behind the greater total porosity for manually mixed BioRoot RCS. 

Considering the regional porosity of the root canal, a higher apical porosity was detected for BioRoot RCS after 7 days and 6 months. Similar results were also reported for lateral compaction using RealSeal sealer (SybronEndo, Orange, CA, USA), but some authors suggested that pores distribution can be unpredictable regardless of the filling method and sealer type [59]. The coronal region of roots filled with GuttaFlow Bioseal and Total Fill BC Sealer was found to exhibit greater initial and long-term porosity than other regions. It can be hypothesized that such a great level of apical porosity in BioRoot RCS could result from the high density of the material, which impaired the placement of air bubbles (creating pores) toward the coronal part of the root during its application. As a consequence, apical porosity and poor seal of the apical area occurred. On the contrary, the great number of coronal porosity of GuttaFlow Bioseal and Total Fill BC Sealer might be induced by the movement of the air bubbles from the apical part to the coronal due to the lower density of these materials. The introduction and gentle pumping movement of the GP cone may have caused a translocation and entrapment of air bubbles within the coronal part of the root. Moreover, the coronal part is the area containing the most sealant, and, therefore, it may undergo volumetric changes during setting and dissolution, contributing to increased porosity. The highest porosity of this region was also found in other studies, regardless of the sealant and the obturation technique used [9,60,61]. It is worth emphasizing that the lack of regional sealing can lead to re-infection and create a critical condition for persistent bacteria accumulation, causing treatment failure. The apical area is probably the most “delicate” and important region within the entire canal system because, during chemo-mechanical shaping, it is the part which is less instrumented and de-contaminated [62]. Consequently, the enhanced penetration of the sealant into this region may trap persistent micro-organisms, providing a hermetic seal of this crucial area.

Some limitations of the present study should be acknowledged. The results of these in vitro evaluations do not reflect phenomena and relations occurring in a real clinical scenario (micro-biome, host immune system, seepage of fluids though accessory canals, temperature changes, occlusal load, root anatomy variations). Thus, clinical studies are necessary to provide a wider perspective on the investigated parameters. Moreover, the limited sample size could have contributed to the high variability of the obtained data and high value of standard deviation. Thus, studies using a larger sample size are recommended. Additionally, only three sealants were used for the study. Therefore, other sealants should be investigated. Moreover, future studies should be performed to evaluate sealants in teeth with complex root anatomy, e.g., greater curvatures, using various techniques of root canal shaping and its obturation including application of thermal methods. 

## 5. Conclusions

Within limitations of the study, it can be stated that:

1. The total volume of pores remained unchanged after 6 months of storage.
2. GuttaFlow Bioseal exhibited significantly higher long-term volume of open pores than Total Fill BC Sealer.
3. The total porosity remained of all investigated sealers unchanged after the 6-month storage except for BioRoot RCS. Total porosity of this materials significantly increased after long-term incubation.
4. Initial total porosity of BioRoot RCS was significantly higher in the apical region than in coronal area.
5. BioRoot RCS exhibited significantly higher initial total porosity in the apical area than GuttaFlow Bioseal.

Therefore, the use of bioactive sealers with excessive tendency to create porosities both at shorth- and long-term period of storage might compromise the long-term success of endodontic treatments. 

## Figures and Tables

**Table 1:** Materials used in this study.

Root Canal Sealer	Manufacturer	Type of the Sealer	Presentation	Composition
GuttaFlow Bioseal	Coltène/Whaledent AG, Altstätten, Switzerland	Silicone-based with calcium silicate	2 pastes in a double syringe with a self-mixing applicator	Gutta-percha powder particles, polydimethylsiloxane, platinum catalyst, zirconium dioxide, calcium silicate, nano-silver particles, coloring, and bioactive glass ceramic
Total Fill BC Sealer	FKG Dentaire, La Chaux-de-Fonds, Switzerland	Calcium–silicate 1 component	Injectable, pre-mixed single syringe	Calcium silicates, calcium phosphate monobasic, zirconium oxide, tantalum oxide, and thickening agents
BioRoot RCS	Septodont, Saint Maur Des Fosses, France	Calcium-silicate 2 components	Manually mixed powder and pre-portioned liquid ampules	Powder: Tricalcium silicate, zirconium oxide, and povidone Liquid: Aqueous solution of calcium chloride and polycarboxylate

**Table 2:** Initial volume and percentage of open and closed pores.

Group	Volume of Open Pores (mm^3^)	Volume of Closed Pores (mm^3^)	Total Volume of Pores (mm^3^)	Percentage Volume of Open Pores (%)	Percentage Volume of Closed Pores (%)
GuttaFlow Bioseal	0.032 ± 0.035	0.175 ± 0.148	0.210 ± 0.150	19.620 ± 22.530	80.380 ± 22.530
Total Fill BC Sealer	0.007 ± 0.015	0.071 ± 0.046	0.078 ± 0.050	7.330 ± 12.190	92.670 ± 12.190
BioRoot RCS	0.036 ± 0.052	0.091 ± 0.088	0.127 ± 0.080	27.120 ± 36.500	72.880 ± 36.500

**Table 3:** Long-term volume and percentage of open and closed pores.

Group	Volume of Open Pores (mm^3^)	Volume of Closed Pores (mm^3^)	Total Volume of Pores (mm^3^)	Percentage Volume of Open Pores (%)	Percentage Volume of Closed Pores (%)
GuttaFlow Bioseal	0.073 ± 0.070 ^A^	0.123 ± 0.159	0.197 ± 0.136	44.370 ± 34.400	55.630 ± 34.400
Total Fill BC Sealer	0.010 ± 0.019 ^A^	0.047 ± 0.033	0.057 ± 0.041	11.340 ± 18.290	88.660 ± 18.290
BioRoot RCS	0.055 ± 0.060	0.113 ± 0.147	0.168 ± 0.160	31.270 ± 31.570	68.730 ± 31.570

Same superscript letters indicate statistically significant differences (*p* < 0.05).

## Data Availability

Not applicable.
